# The consistent anti-cancer effect of a simple exercise (Ou MC decrescendo phenomenon exercise) may hold promise for low-cost cancer prevention

**DOI:** 10.1097/MS9.0000000000001824

**Published:** 2024-02-16

**Authors:** Ming Cheh Ou, Fu Min Chen

**Affiliations:** a Department of Obstetrics and Gynecology, Zhong-Xiao Branch, Taipei City Hospital; b Department of Obstetrics and Gynecology, Chung San Hospital, Taipei City, Taiwan, ROC

**Keywords:** anti-cancer effect, cancer prevention, Ou MC decrescendo phenomenon exercise, physical activity

## Abstract

The causal relationship between physical activity and anti-cancer effect are not proved by the current studies. However, Ou MC decrescendo phenomenon treatment (OuDPt), a simple exercise treatment, has shown consistent anti-cancer effects, which evinces the consequent anti-cancer effect by physical activity. The anti-cancer effects through OuDPt in the context of physical activity and human body anatomical axes showed to induce apoptosis, restore apical-basal polarity of cancer cells and mitigate epithelial-mesenchymal transition (EMT) with concomitant clinical regression of uterine endometrial cancer, suppression of ovarian and pancreatic cancer growth, regression of early suspicious pancreatic cancer, enhancement of chemotherapy effect of pancreatic cancer and cessation of cancer-related bleeding, which underlines the most important anti-cancer mechanisms. Although such anti-cancer effects by OuDPt show insufficient efficacy for advanced cancer in long-term treatment, OuDPt may be availed as an Ou MC decrescendo phenomenon exercise for cancer prevention. Further study is warranted.

## Introduction

Highlights

- Ou MC decrescendo phenomenon treatment (OuDPt), a simple exercise treatment, has shown consistent anti-cancer effects, which evinces the consequent anti-cancer effect by physical activity and may contribute to cancer prevention.
- It has been found that prevention with treatment before a diagnosis is the best option for cancer prevention (Akhmetzhanov and Hochberg, 2015, ref^1^).
- Thus, the OuDPt, a simple exercise treatment, can be used preventively before the cancer occurs or grows as an exercise—Ou MC decrescendo phenomenon exercise (OuDPe).
- OuDPe can be performed by people themselves and may hold promise as a low-cost cancer prevention method.

Physical activity has been associated with reduced cancer risk in many studies. However, evidence linking higher physical activity to lower cancer risk comes mainly from observational studies, and a causal relationship has not been proven^2^. Ou MC decrescendo phenomenon treatment (OuDPt) is a simple exercise treatment and has shown consistent anti-cancer effects, which is a solid evidence for the anti-cancer effects by physical activity^3^. OuDPt is mainly self-administered by the patient with placing the contralateral hand along human body anatomical axes (HBAAs) over a diseased location to produce a zone with less pain or inflammation under the hand^4^. Ou MC decrescendo phenomenon (OuDP) appears to be consistently effective for treating a wide variety of diseases in terms of distance between hand and lesion, treatment duration and performance frequency. The effect of OuDP can be significantly increased by decreasing the distance between hand and lesion, increasing treatment duration, and raising treatment frequency^5^. Prompt remission of joint pain, oedema of soft tissue by trauma, pain by infection, cessation of uterine bleeding by cancer or cancer regression in the studies with OuDP indicate a restoration of normal tissue function^3,5^. Restoration of normal tissue function may re-establish host defense systems, which will contribute to defense against microorganisms, inflammation, degenerative changes, and cancers. The normalization of function of cancer cells may make them conform to the regulations by apoptosis, growth suppression, and metastatic hindrance that undergo with normal cells^6^. The normalization of tissue function through OuDPt apparently not only involves the tumour cell but also the microenvironment in which tumour cells lodge^7^. The tumour microenvironment has been regarded as a main factor for tumour development and metastasis. A normalization of tumour microenvironment will place tumour cells under the circumstances that suppress metastasis, prevent uninhibited proliferation, minimize angiogenesis and eliminate abnormal cells with normalized host immunological systems^6^.

### Clinical investigation of the mechanism of Ou MC decrescendo phenomenon

#### Human body anatomical axes interactions effect

The mechanisms involved in mutual interactions between bilateral parts of topographically symmetrical living beings remains largely unknown, but there are indications that it may occur via neurological transmission^8^. Studies have shown that such interactions may induce and reinforce inflammatory reactions and pain sensations on the contralateral side of the body. Examples of such phenomena are contralateral arthritis induced by unilateral arthritis in rats and, symmetrically developed arthritis, pulmonary fibrosis, glomerulonephritis and sympathetic ophthalmia in humans^8^. The Ou MC manipulation method results in the OuDP in abdominal palpation, which involves the alleviation of pain by the placement of the contralateral hand of the examiner on the acute abdomen of the female patient^9^. A front study of Ou MC manipulation method (2006) compared the effect of contralateral to ipsilateral hands of an examiner when used to alleviate the acute abdomen pain of 42 women. In this case, usage of the contralateral hand alleviated the pain of 92.3% of women (39/42), while the ipsilateral hand did not (0/42) (*P*<0.001, Paired *t*-test)^5^. For another 14 patients self-administered OuDPt, performing with the ipsilateral hand showed effective for only one patient while OuDPt performed with the contralateral hand showed effective for all the 14 patients (*P*<0.001, two-sided, McNemar test)^10^. OuDPt usually show measurable immediate effects when relieving clinical symptoms of patients, which indicates a prompt tissue functional normalization to slow down the inflammatory process.

Studies have shown that inflammation on one side of a topographically symmetrical living organisms may cause further inflammation on the contralateral side, which indicates an interaction occurring in relation to HBAAs^8^. However, the OuDPt invokes a restorative effect to the opposing side of the body rather than a spread of the harmful effects of inflammation. A similar phenomenon to OuDP was described in the studies of the snapping shrimp where it was found that the contralateral snapper claw of the snapping shrimp affects the development of the new claw after the claw is lost in order to achieve a bilateral symmetry in both shape and fibre composition of bilateral claw^11^. Interestingly, the exposure to conspecific mutual interactions of other snapping shrimps also triggers such claw transformation^11^. Similarly, the OuDP can be invoked a human body response using both self and therapist administration. The signals are imparted during such exposure to such interaction remains to be elucidated.

#### Multi-dimensional enhancement effect

The three dimensional (3D) tissue organization model suppressing the traits of cancer cells more effective than 2D model has been hypothesized where normal cells use their internal cell polarity mechanisms to establish a polarized 3D tissue organization, which, in turn, uses the apical junctional complexes and cell-substratum adhesions to reinforce, maintain polarity and thus normalize the mutant cells with disrupted internal cell polarity pathways to conform to the regulations by apoptosis, growth suppression, and metastatic hindrance that undergo with normal cells^12^. Similarly, the interactions among multiple HBAAs with OuDPt also render a 3D treatment model that is more effective than a 2D treatment model (among two HBAAs)^13^. However, our studies show OuDPt may probably directly normalize the dysfunctional cells than indirectly through the reinforcement by the neighbouring normal cells for that OuDP shows to render immediate effects on pain, inflammation and uterine neoplasm bleeding, which is too fast to consider as an indirect effect^13^.

Our study has shown that OuDPt along the 3D human body polarity system composed of left-right, dorsoventral and vertical HBAAs suppressed neoplasm development more efficiently than OuDPt along the 2D polarity body system, providing evidence that the effect of OuDPt is associated with interaction of HBAAs^13^. The interactions among multiple HBAAs in OuDPt may renders a 3D organization model that, similar to a potential non-canonical tumour suppressor that prevents the manifestation of neoplastic features in mutant cells and, ultimately, suppresses tumour development and progression^12^.

#### Normalization of cell polarity

**Figure 1:**
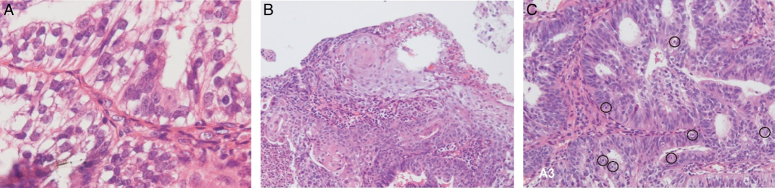
Endometrial biopsies of the patient with uterine endometrioid carcinoma IIIB before and after Ou MC decrescendo phenomenon treatment (OuDPt) only treatment. (A): Biopsy before OuDPt showed grade 1 endometrioid carcinoma with epithelial-mesenchymal transition (EMT), loss of epithelial apical-basal polarity, and no definite squamous metaplasia or apoptosis can be identified [hematoxylin and eosin (HE), magnification 400×]. (B), (C): Endometrial biopsy after 29 days of OuDPt treatment. (B) showed prominent squamous differentiation of tumour cells. (C) showed numerous apoptotic cells (black circles) with restored apical-basal polarity of tumour cells and diminution of EMT (HE, magnification 100×). (Photo courtesy of Ou MC *et al.* ^5^).

An endometrial cancer stage IIIB of a 49-years-old woman with both cervical and endometrial biopsies showed grade 1 endometrioid carcinoma with prominent epithelial-mesenchymal transition (EMT), loss of epithelial apical-basal polarity, and no definite squamous differentiation or apoptosis can be identified (Fig. 1A). The endometrial biopsy following 29 days of OuDPt showed tumour cells with prominent apoptosis changes, squamous differentiation, restored apical-basal polarity with diminution of EMT, which indicated normalization of polarity of tumour cells (Fig. 1B, C). The endometrial cancer of this patient regressed from clinical stage IIIB to IA by MRI (Magnetic resonance imaging) after 5 months of OuDPt. During the therapeutic period, this case did not receive any other anti-cancer treatment except OuDPt^7^.

Restored apical-basal polarity and diminution of EMT of aberrant cancer cells indicates a normalization of cell polarity from cancer by OuDPt^14^. The tumour regression by OuDPt may indicate that disruption of cell polarity is not only a by-product of cancer cells but also plays a causal role of cancer initiation and development.

#### Normalization of tissue function

OuDPt appears to be consistently effective based on the distance between the hand and the lesion, on the duration of the treatment and on the frequency of performance. The potential roles of several sensory factors merit exploration for the mechanism of action of OuDPt. When OuDPt is performed, the area of treatment not only perceives the performing hand by proprioception or touch, but also by temperature change as well. Elevation of tissue temperature by thermal therapy has been shown to increase cell-mediated cytotoxicity, which may have anti-bacterial, anti-parasitic, anti-viral, and anti-neoplastic effects when treating dermatological diseases^15^. However, two findings argue against the involvement of thermal effects for OuDPt. Firstly, a patient with cellulitis in a study responded to OuDPt despite not benefitting from far infrared ray therapy. Secondly, when the ambient temperature during treatment is room temperature or below, the hand is usually cooler than the body while performing OuDPt^5^. Touch may also not be essential to the effectiveness of the OuDPt for the OuDPt does not require any hand movement or even skin contact when treating lesions though as close as possible^5,16^.

The recovery of tissue by OuDPt from diseases unrelated to inflammation indicates that a restoration of normal tissue function seems to be occurring after administration of OuDPt^5^. Restoration of normal tissue function may involve re-establishing host defense systems, which contribute to defense against microorganisms, inflammation, degenerative changes, and cancers^17^. However, the restoration of normal tissue function provided by the initial treatment may be inadequate to cure the disease, as was evidenced by the finding that many patients required repeated sessions of OuDPt to yield a long-term effect^5,10^. The patients with OuDPt failure to give persistent relief for infection suggests that restored tissue function may not be always sufficient to cure an infection^10^. However, OuDPt can result in prompt remission of joint pain, reduction of oedema of soft tissue caused by trauma, reduction in pain caused by infection, recovery for degenerative changes, cessation of uterine bleeding or cancer regression, which indicates the restoration of normal tissue function^3^.

### Physical activity and cancer prevention

Physically active adults have a significantly lower risk of developing several commonly occurring cancers, as well as lower risk of several other cancers. Studies show that adults who participate in physical activity have strong evidence of reduced risks of developing cancers of the breast, colon, endometrium, bladder, stomach, oesophagus (adenocarcinoma) and kidney, and moderate evidence for an association with lung cancer risk, with 10–20% reductions in relative risks^18^.

Exercise is postulated to reduce tumour development through multiple mechanisms such as the followings: (a) vascularization and perfusion, (b) immune function, (c) tumour metabolism, and (d) muscle–cancer interaction^19^. However, the cause–effect relationship between these mechanisms and the control of tumour genesis and growth are not established such that the potential interrelationship between exercise and cancer remains still unclear^2^.

Nonetheless, OuDPt has shown a consistent cause–effect anti-cancer effect and evinces the causal relationship of anti-cancer effect with physical activity, which is similar to that between antibiotics and bacterial infection^3^. The anti-cancer effects by OuDPt in the context of physical activity and human body anatomical axes showed to induce apoptosis, squamous differentiation, cancer cells with restored apical-basal polarity and diminution of EMT with concomitant regression of uterine endometrial cancer, suppression of ovarian and pancreatic cancer growth, regression of early suspicious pancreatic cancer, enhancement of chemotherapy effect of pancreatic cancer and cessation of cancer-related bleeding^3^. OuDPt shows a causal relationship between physical activity and anti-cancer effects, which demonstrates normalization of cell polarity and tissue function, regression of cancer in the context of physical activity and human body anatomical axes.

### Ou MC decrescendo phenomenon exercise (OuDPe)

OuDPt is a simple exercise treatment and demonstrates anti-cancer effects on cancer diseases such as endometrial cancer, pancreatic cancer, uterine leiomyosarcoma, and ovarian cancer^3^. OuDPt has shown to normalize cell polarity and tissue function of cancer in the context of physical activity and human body anatomical axes, which underlines the most important anti-cancer mechanisms and may contribute to cancer prevention^3^. Most patients required repeated sessions of OuDPt to yield a long-term effect, which indicates the duration, intensity and frequency of OuDPt is related to the effect of treatment. The most effective distance of OuDPt to a lesion has been estimated less than 0.5 cm and the more prominent cancer regression by OuDPt with a shorter distance, which makes it less effective for greater lesions^20^. Thus, the anti-cancer effects of OuDPt are associated with factors such as the frequency, duration, and intensity of treatment, as well as the accessibility and susceptibility of the tumour. This relationship mirrors the dynamics between antibiotics and bacterial infections, where similar factors come into play.

A mathematical model was availed by Akhmetzhanov and Hochberg to compare cancer prevention with treatment before a diagnosis and cancer intervention with treatment after a diagnosis^1^. In many cases, it was found that prevention with treatment before a diagnosis is the best option. The preventative treatment strategy is often significantly better than the post-diagnostic treatment strategy, both in extending the tumour-free life of patients and in reducing the chance of treatment failure^21^. It indicates the exercise with OuDPt can be used preventively before the cancer occurs or grows as an OuDP exercise (OuDPe).

**Figure 2:**
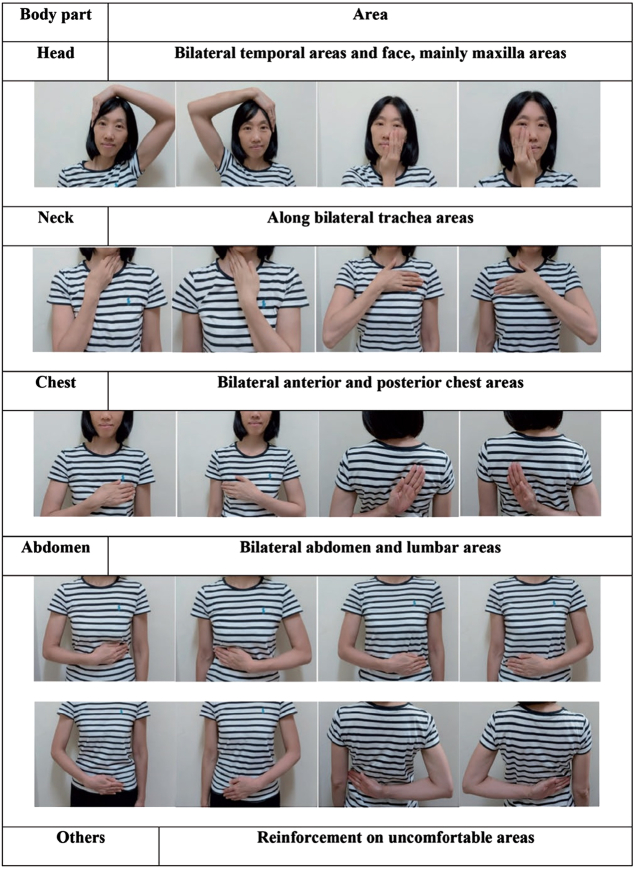
A model of Ou MC decrescendo phenomenon exercise with multiple human body anatomical axes interactions for each possible cancer site. (Permission from Ou MC *et al.* ^22^).

A model of OuDPe is demonstrated in Figure 2 to perform for each possible cancer site^22^. The frequency, duration, intensity for OuDPe to be formulated may depend on the physical condition of different people. More detailed recommendations of OuDPe for cancer prevention warrants further study.

## Conclusion

The currently available data provide substantial evidence that the effect of exercise may predict a stronger association with cancer and could supplementarily be introduced in the cancer clinical practice to improve disease progression and prognosis^2,18^. Findings from observational studies provide much evidence for a link between higher levels of physical activity and lower risk of cancer^2^. However, these studies cannot fully rule out the possibility that active people have lower cancer risk because they engage in other healthy lifestyle behaviours.

However, OuDPt shows a consistent cause–effect anti-cancer effect and evinces the causal relationship of anti-cancer effect with physical activity, which is similar to that between antibiotics and bacterial infection^3^. It has been found that the preventative treatment strategy is often significantly better than the post-diagnostic treatment strategy on cases of cancer; thus, prevention with treatment before a diagnosis is the best option for cancer prevention^1,21^. OuDPe (Fig. 2) is a simple preventive OuDPt exercise and can be done by people themselves. OuDPe may hold promise as a low-cost cancer prevention method. However, further study is warranted.

## Ethical approval

Ethics approval is not required for this conference report.

## Consent

Informed consent is not required for this conference report.

## Source of funding

Not applicable.

## Author contribution

All authors have made substantial contributions and given final approval of the version to be published.

## Conflicts of interest disclosure

Not applicable.

## Research registration unique identifying number (UIN)

Research registration is not required for this conference report according to Taiwan Regulations on Human Trials. However, we confirm that any aspect of the collected studies covered in this report have been conducted with the ethical approval of all relevant bodies.

## Guarantor

All authors are in full responsibility for the work, the conduct of the study and the access to the data, and controlled the decision to publish.

## Data availability statement

All data in this article are publicly available.

## Provenance and peer review

Not commissioned, externally peer-reviewed.

## Presentation

This paper was presented in 2nd JCA-AACR precision cancer medicine international conference. Kyoto, Japan, June, 2023 and IGCS annual meeting, Seoul, Korea, November, 2023.
